# Doctors' Fees and Nursing Homes

**Published:** 1912-08-17

**Authors:** 


### The Hospital

The Workers' Newspaper of Administrative Medicine and Institutional Life, Administration, National Insurance and Health.

No. 1363, Vol. LII. SATURDAY, AUGUST 17, 1912. PRICE ONE PENNY.

### DOCTORS' FEES AND NURSING HOMES.

At this season of the year when many busy men find themselves for once in a way with leisure for contemplation of subjects outside their own spheres of work and activity, the private nursing home as it at present flourishes amongst us often comes in for criticism in the correspondence columns of the public press. We should be the last to deny that the existing system, conduct, and charges of private nursing homes are free from abuses and defects. Indeed the pressing need of many reforms has been persistently urged in these columns for some years; and we feel entitled to claim that certain improvements which recent years have witnessed have been along lines first suggested by The Hospital.

By no means blind worshippers of the existing conditions, we nevertheless believe that the average standard is being raised, even though slowly. In our last issue (The Hospital, August 10, 1912, p. 498) an Anglo-Indian correspondent entered an eloquent plea for the private nursing home and those employed in it. The critical might reply that "M. E. B.'s" defence was rather a rhapsody of the ideal than a description of the actual. But, after all, it is not given to many of us to reach an ideal standard; and many people hold that an attainable ideal is not an ideal worth following at all. At least it is a sign of grace to cherish such an ideal; and if nursing homes are attracting the kind of woman whose praises our correspondent sang, then it cannot be denied that they are improving.

So far this August, the voice of the critic of nursing homes has not reached us; but an eponymous letter-writer to the *Times* has made complaint of certain matters which touch the association of consulting physicians and nursing home proprietors. This complainant, who signs himself "Fair Game," sets forth a plea which may be summed up thus. He was advised by his physician to enter a nursing home for a course of treatment, consisting mainly of rest in bed and massage, which lasted rather more than six weeks. During this time he was visited three times a week by the doctor, who saw him in all twenty-one times and charged him forty-two guineas. "Fair Game's" opinion is that one visit a week would have been ample for the needs of his case, and that the doctor had a regular routine of seeing all his patients in that home each time he called there, irrespective of their needs and condition.

The essence of the complaint is, therefore, seen to be that unnecessary visits were paid to him merely because the doctor had other patients in the same building, and because thus he could make more fees. Since no particulars are given of the nature of the illness, it is not easy to judge whether there is substantial foundation for this allegation. Certainly there are men in the profession, and in the purlieus of Harley Street, who are quite capable of these despicable actions; that is regrettable, even disgraceful, but it is not a crime for which either the profession at large or the consulting sections of it can be in bulk convicted. The fact that there are such mercenary and dishonourable physicians is one, but of course only one, of the many good reasons why a patient who wants to see a consultant is well advised to take the opinion of his general practitioner as to whom to consult; for straight dealing with himself and the patient is one of the qualifications which the experienced practitioner most appreciates in a physician or surgeon. If "Fair Game's" suspicions be correct, it must be admitted that he has fallen into the hands of a dishonest man; and since the milk is now spilt, the only thing to do is to make a mental note of the fact for future reference. There is, however, a possibility which has not struck him. Possibly the three visits a week really were required by the exigencies of his case; possibly he stood in graver danger than he has any idea of; possibly he got full value for his forty-two guineas. The particulars he gives are so vague that there are no means of judging whether these suppositions are true or not; we merely suggest them as at least feasible.

In any case, whether "Fair Game" was or was not hardly used by his physician, his final paragraphs leave us cold. For therein he attacks the nursing-home system for the faults (as he sees them) of a doctor. He denounces the "system which enables a doctor to keep you for prolonged periods in a nursing home of his own choosing. . ." Here he really tilts at windmills. For there is no such "system"; and no doctor can "keep" any sane patient against his will in any home for six seconds, let alone for six weeks.

The physician, indeed, only "chooses" the home in the sense that he recommends it as a well-conducted institution to a patient who wants to be advised where he should go. On this flimsy score the writer apparently seeks to discredit both nursing homes and the medical profession. We sympathise with his resentment at the misconduct which he believes he experienced from his physician; but our sympathy is withheld when he proceeds to hasty and unsound generalisations based upon misrepresentations and distorted statements of fact. If this were the worst evil that could be alleged in it, we should be well content with the nursing-home system.

